# The oribatid mite genus *Papillocepheus* (Acari, Oribatida, Tetracondylidae), with description of a new species from southern Vietnam

**DOI:** 10.3897/zookeys.381.6832

**Published:** 2014-02-18

**Authors:** Sergey G. Ermilov, Alexander E. Anichkin, Andrei V. Tolstikov

**Affiliations:** 1 Tyumen State University, Tyumen, Russia; 2 A.N. Severtsov Institute of Problems of Ecology and Evolution, Russian Academy of Sciences, Moscow, Russia; 3 Joint Russian-Vietnamese Tropical Research and Technological Center, Hanoi-Ho Chi Minh, Vietnam

**Keywords:** Oribatid mites, new species, description, *Papillocepheus*, generic diagnosis, new record, key, Vietnam

## Abstract

The genus *Papillocepheus* is recorded in the Oriental region for the first time. A new species, *Papillocepheus primus* **sp. n.**, is described from southern Vietnam; the description is based on specimens collected from semidecayed leaves and litter of Dong Nai Biosphere Reserve and Bu Gia Map National Park. The new generic diagnosis of *Papillocepheus* and an identification key to the known species of this genus are given.

## Introduction

*Papillocepheus* (Acari, Oribatida, Tetracondylidae) is the genus of oribatid mites that was proposed by Balogh and Mahunka (1966) with *Papillocepheus heterotrichus* Balogh & Mahunka, 1966 as type species. Currently, this genus comprises nine species, which are collectively distributed in the Ethiopian and Australian regions, Yemen and Chile. These species are as follows: *Papillocepheus areolatus* Mahunka, 1987 (Mahunka 1987; recorded from Kenya), *Papillocepheus decoratus* Mahunka, 1994 (Mahunka 1994; Madagascar), *Papillocepheus decorus* (Hammer, 1966) (Hammer 1966; New Zealand), *Papillocepheus deficiens* J. & P. Balogh, 1983 (Balogh and Balogh 1983; Australia); *Papillocepheus heterotrichus* Balogh & Mahunka, 1966 (Balogh and Mahunka 1966; South Africa), *Papillocepheus longisetosus* Mahunka, 2009 (Mahunka 2009; Yemen), *Papillocepheus neotropicus* (P. Balogh, 1988) (Balogh and Balogh 1988; Chile), *Papillocepheus reductus* Mahunka, 1994 (Mahunka 1994; Madagascar) and *Papillocepheus tuberculatus* (Mahunka, 1978) (Mahunka 1978; Mauritius).

In the course of taxonomic identification of Vietnamese oribatid mites collected in October and November 2013 we found one new species, belonging to the genus *Papillocepheus*. Hence, the genus is recorded in Vietnam and the Oriental region for the first time. The main purpose of our paper is to describe and illustrate this species.

Also, the new generic diagnosis of *Papillocepheus* and an identification key to the known species of this genus are provided.

## Material and methods

Specimens of *Papillocepheus primus* sp. n. were collected by A.E. Anichkin and S.G. Ermilov in southern Vietnam. Holotype, female: Dong Nai Province, Dong Nai Biosphere Reserve, 11°26'N, 107°26'E, 120 m a.s.l., semidecayed leaves of the Moracea family in a monsoon semideciduous tropical forest on sandy soils near (0.5 m) Dong Nai river, 25.X.2013. Paratype, female: Binh Phuoc Province, Bu Gia Map National Park, 12°11'N, 107°12'E, 539 m a.s.l., leaf litter on ferralitic soils (sifting) in Palm forest on slope of a hill near small river, 14.XI.2013.

All specimens were studied in lactic acid, mounted in temporary cavity slides for the duration of the study, and then stored in 70% ethanol in vials. Body measurements are presented in micrometers. The body length was measured in lateral view, from the tip of the rostrum to the posterior edge of the ventral plate. Notogastral width refers to the maximum width in dorsal aspect. Lengths of body setae were measured in lateral aspect. Formulae for leg setation are given in parentheses according to the sequence of trochanter–femur–genu–tibia–tarsus (famulus included). Formulae for leg solenidia are given in square brackets according to the sequence of genu–tibia–tarsus. Terminology used in this paper mostly follows that of Norton and Behan-Pelletier (2009).

## Systematics

### Genus Papillocepheus

Balogh & Mahunka, 1966

http://species-id.net/wiki/Papillocepheus

Clavazetes Hammer, 1966

#### Type species.

*Papillocepheus heterotrichus* Balogh & Mahunka, 1966

#### New generic diagnosis

(based partially on data from: Balogh and Mahunka 1966; Hammer 1966).

Tetracondylidae with the following combination of characters: costulae dorsal or dorso-lateral, reach the insertions of lamellar setae; transcostula present or absent; rostral, lamellar and interlamellar setae well developed, setiform or weakly dilated distally or medio-distally; sensilli with short stalk and clavate head; exobothridial setae absent; notogaster with 8–10 pairs of setae, all medium size or short; majority of notogastral setae clearly dilated in distal or medial part, phylliform or willow leaf shaped; medial prodorsal and notogastral condyles usually absent, when present, separated; epimeral formula 3–1–3–3, sometimes some setae absent or represented by alveoli; genital plates with three setae; aggenital setae present or absent; anal plates with two pairs of setae; three pairs of adanal setae present; adanal setae *ad*_3_ located in lateral or preanal position; localization of adanal lyrifissures different among types; setae *u* of all leg tarsi setiform.

### Papillocepheus primus

Ermilov, Anichkin & Tolstikov sp. n.

http://zoobank.org/CB231C4F-387C-46BE-9C54-0A71399761AD

http://species-id.net/wiki/Papillocepheus_primus

Figs 1 –5

#### Diagnosis.

Body size 498 × 273–282. Rostral setae simple, barbed; lamellar setae shorter, thickened, barbed; interlamellar setae thick, willow leaf shaped, densely barbed. Sensilli with barbed head. Medial prodorsal and notogastral condyles present, notogastral ones located close to each other; lateral prodorsal and notogastral condyles absent. Notogaster with 10 pairs of phylliform setae. Epimeral setal formula: 3–1–3–3. Anal setae dilated in medial part. Adanal *ad*_1_, *ad*_2_ phylliform; *ad*_3_ slightly thickened in medial part, inserted in lateral position. Adanal lyrifissures located in paraanal position, distanced from the anal plates. Most setae on leg tarsi smooth, with swelling in tip.

#### Description.

*Measurements*. Body length 498 (holotype and paratype: both female); body width 273 (holotype), 282 (paratype).

*Integument*. Body color yellow-brownish. Body surface and legs covered by granular cerotegument; granules conical (length up to 4). Body surface (including genital and anal plates) densely microfoveolate (diameter of foveolae up to 1). Lateral parts of prodorsum, notogaster and anogenital region additionally with larger foveolae (diameter of foveolae up to 6). Lateral region of body near to pedotecta II and anterior margin of notogaster partially tuberculate (diameter of tubercles up to 8).

*Prodorsum*. Rostrum simple, widely rounded. Costulae well developed, thin. Transcostula absent. Rostral setae (*ro*, 69–77) setiform, barbed, inserted laterally. Lamellar setae (*le*, 49–57) shorter, slightly thicker and more densely barbed than rostral setae, inserted dorso-laterally near the end of costulae. Interlamellar setae (*in*, 77–86) thick, willow leaf shaped with attenuate tip, densely barbed. Sensilli (*ss*, 32–36) short, with barbed head. Medial prodorsal condyles (*co.pm*) small, rounded distally. One indistinct tubercle located laterally to each medial condyle, possibly, it is the second pair of medial prodorsal condyles. Lateral prodorsal condyles absent. Distinct tutorial lines absent.

*Notogaster*. Medial notogastral condyles (*co.nm*) of medium size, weakly triangular distally, located close to each other, between prodorsal medial condyles. Lateral notogastral condyles absent. Notogaster with 10 pairs of notogastral setae. All setae widely phylliform; *c* (45–49) longer than others (32–36). Opisthonotal gland openings and lyrifissures *ip*, *ih*, *ips* poorly visible.

*Gnathosoma*. Subcapitulum longer than wide: 123 × 94. Subcapitular setae setiform, smooth; *h* and *m* (both 57) longer than *a* (24). Adoral setae absent. Palps (length 82) with setation 0–2–1–3–8(+ω). Solenidion thickened, blunt-ended, pressed to the palptarsus surface, not attached with eupathidium. Chelicerae (length 139) with one barbed seta *cha* (45); seta *chb* not evident. Trägårdh’s organ conical.

*Lateral podosomal and epimeral regions*. Epimeral setal formula: 3–1–3–3. All setae setiform, smooth. Setae *1b*, *3b* (57–61) longer than other setae (36–41). Discidia (*dis*) rounded.

*Anogenital region*. Three pairs of genital (*g*_1_–*g*_3_, 18–20) and one pair of aggenital setae (*ag*, 32–36) setiform, smooth. Two pairs of anal setae (*an*_1_, *an*_2_, 18–20) thickened, dilated in medial part, barbed. Three pairs of adanal setae present: *ad*_1_, *ad*_2_ (16) phylliform, inserted in postanal position; *ad*_3_ (16–18) slightly thickened in medial part, barbed, inserted in lateral position. Adanal lyrifissures *iad* located in paraanal position, distanced from the anal plates.

*Legs*. Generally, morphology of leg segments typical for Tetracondylidae (Grobler 1995; Ermilov et al. 2010). Claw of each tarsus smooth. Tarsi without teeth. Formulae of leg setation (including famulus) and solenidia: I (1–4–3–4–16) [1–2–2], II (1–4–3–3–15) [1–1–2], III (2–3–0–2–14) [1–1–0], IV (1–2–1–2–13) [0–1–0]; homology of setae and solenidia indicated in Table 1. Most setae on tarsi smooth, with swelling in tip. Other setae setiform, barbed (except *v*’’ on tibia IV, dilated distally and densely barbed). Seta *ft*’ absent on tarsi III, *ft*’ present on tarsi IV. Seta *l*’ absent on genua III, IV. Famulus short, straight. Solenidia simple.

**Table 1.** Leg setation and solenidia of adult *Papillocepheus primus* sp. n.

Leg	Trochanter	Femur	Genu	Tibia	Tarsus
I	*v*’	*d*, (*l*), *bv*’’	(*l*), *v*’, σ	(*l*), (*v*), φ_1_, φ_2_	(*ft*), (*tc*), (*it*), (*p*), (*u*), (*a*), *s*, (*pv*), *e*, ω_1_, ω_2_
II	*v*’	*d*, (*l*), *bv*’’	(*l*), *v*’, σ	*l*’, (*v*), φ	(*ft*), (*tc*), (*it*), (*p*), (*u*), (*a*), *s*, (*pv*), ω_1_, ω_2_
III	*l*’, *v*’	*d*, *l*’, *ev*’	σ	(*v*), φ	*ft*’’, (*tc*), (*it*), (*p*), (*u*), (*a*), *s*, (*pv*)
IV	*v*’	*d*, *ev*’	*d*	(*v*), φ	(*ft*), (*tc*), (*p*), (*u*), (*a*), *s*, (*pv*)

Roman letters refer to normal setae (*e* to famulus), Greek letters to solenidia. Single prime (’) marks setae on anterior and double prime (’’) setae on posterior side of the given leg segment. Parentheses refer to pair of setae.

**Figures 1–2.**
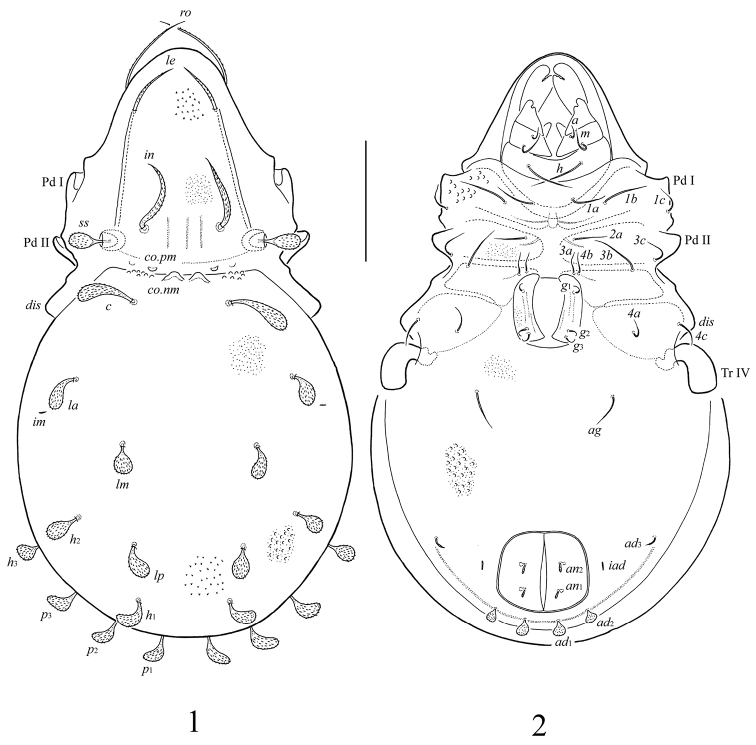
*Papillocepheus primus* sp. n., adult: **1** dorsal view **2** ventral view (legs except trochanters IV not illustrated). Scale bar 100 μm.

**Figures 3–5.**
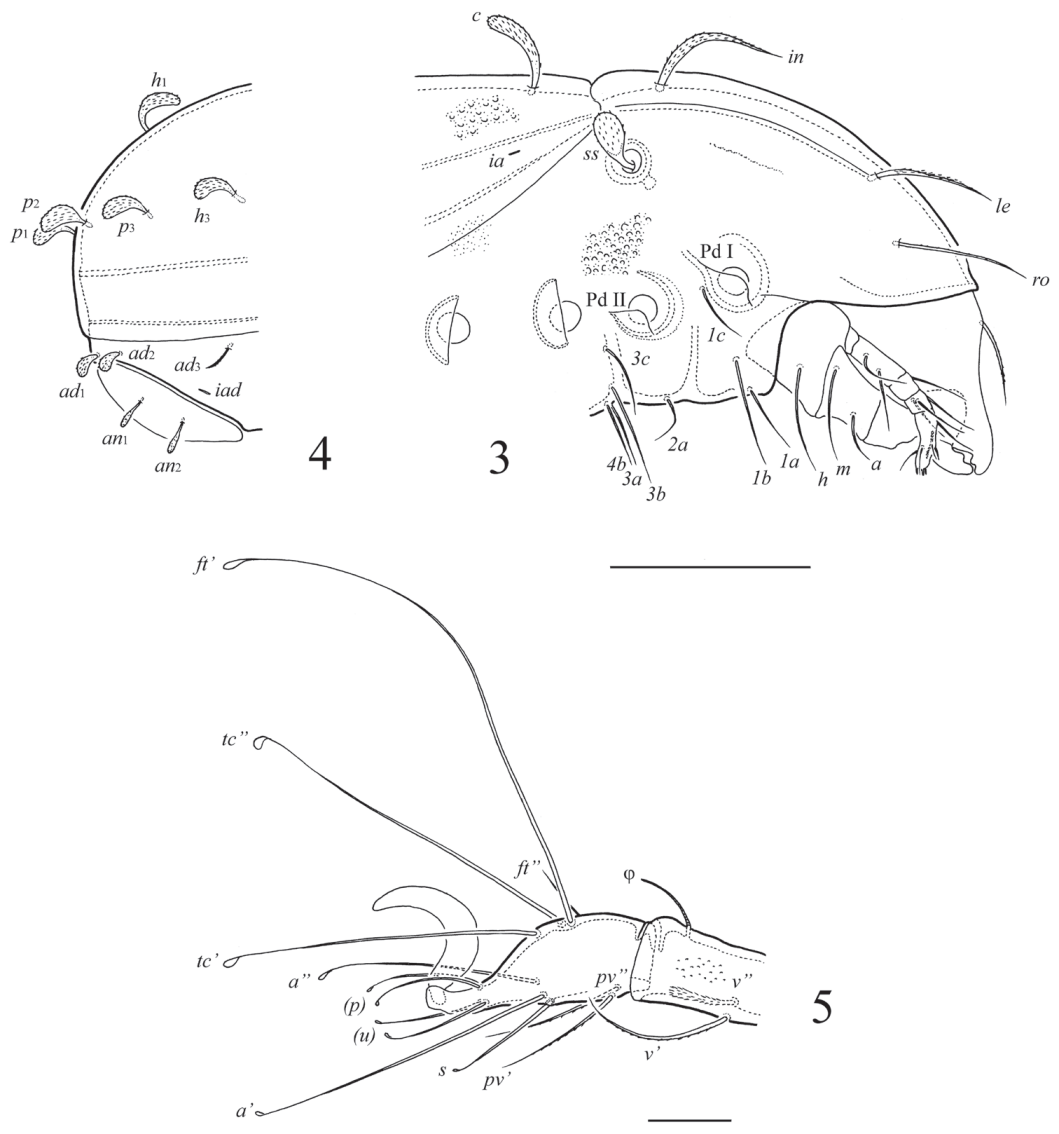
*Papillocepheus primus* sp. n., adult: **3** lateral view of prodorsum and anterior part of notogaster (legs not illustrated) **4** lateral view of posterior part of notogaster **5** tarsus and anterior part of tibia of leg I, right, antiaxial view. Scale bar (**3, 4**) 100 μm, (**5**) 20 μm.

#### Type deposition.

The holotype is deposited in the collection of the Zoological Institute of the Russian Academy of Sciences, St. Petersburg, Russia; one paratype is in the collection of the Tyumen State University Museum of Zoology, Tyumen, Russia.

#### Etymology.

The specific name “*primus*” refers to the first species of *Papillocepheus* recorded in the Oriental region.

#### Comparison.

*Papillocepheus primus* sp. n. can be distinguished from all known species of the genus *Papillocepheus* by the key, which is presented below.

### Key to known species of the genus *Papillocepheus*

**Table:** 

1	Eight or nine pairs of notogastral setae present	2
–	Ten pairs of notogastral setae present	3
2	Eight pairs of notogastral setae present (*c* and *h*_1_ absent); notogastral setae *la*, *lm*, *lp*, *h*_2_ well dilated distally, phylliform, setae *p*_1_–*p*_3_, *h*_3_ simple; setae *lm* located posterior to *la*; adanal lyrifissures in direct apoanal position; body size: 565–620 × 206–261	*Papillocepheus reductus* Mahunka, 1994
–	Nine pairs of notogastral setae present (*c* absent); all notogastral setae weakly dilated in medial part, willow leaf shaped; *lm* located medio-posterior to *la*; adanal lyrifissures almost transversely oriented; body size: 503 × 230	*Papillocepheus deficiens* J. & P. Balogh, 1983
3	Medial prodorsal or/and medial notogastral condyles developed	4
–	Medial prodorsal and medial notogastral condyles not developed	6
4	Translamella present; *lm* located posterior to *la*; aggenital setae absent; adanal lyrifissures in preanal position; body size: 582 × 290	*Papillocepheus longisetosus* Mahunka, 2009
–	Translamella absent; *lm* located medio-posterior to *la*; aggenital setae present; adanal lyrifissures in paraanal position	5
5	Medial notogastral condyles not developed; adanal setae *ad*_3_ in preanal position; adanal lyrifissures located close to the anal plates; body size: 436–471 × 202–224	*Papillocepheus tuberculatus* (Mahunka, 1978)
–	Medial notogastral condyles developed; adanal setae *ad*_3_ in lateral position; adanal lyrifissures distanced from the anal plates; body size: 498 × 273–282	*Papillocepheus primus* sp. n.
6	Notogastral setae *lm* located dorsally on notogaster, medio-posterior to *la*; aggenital setae present	7
–	Notogastral setae *lm* located dorso-laterally on notogaster, posterior to *la*; aggenital setae absent	8
7	Adanal setae *ad*_1_, *ad*_2_ simple; aggenital setae located closer to genital plates than to anal plates; body length: 470	*Papillocepheus neotropicus* (P. Balogh, 1988)
–	Adanal setae *ad*_1_, *ad*_2_ dilated distally, phylliform; aggenital setae halfway between genital and anal plates; body length: 500	*Papillocepheus decorus* (Hammer, 1966)
8	Notogastral setae *c* minute, thin; notogastral setae *p*_1_–*p*_3_, *h*_3_ similar in size to other notogastral setae (except *c*); adanal setae simple; body size: 715–720 × 353–358	*Papillocepheus heterotrichus* Balogh & Mahunka, 1966
–	Notogastral setae *c* well developed, phylliform; notogastral setae *p*_1_–*p*_3_, *h*_3_ smaller than other notogastral setae (except *c*); some adanal setae phylliform	9
9	Translamella present; interlamellar setae straight, weakly dilated distally; adanal lyrifissures longitudinal oriented; body size: 482–517 × 221–266	*Papillocepheus decoratus* Mahunka, 1994
–	Translamella absent; interlamellar setae curved, willow leaf shaped; adanal lyrifissures transversely oriented; body size: 549–590 × 246–279	*Papillocepheus areolatus* Mahunka, 1987

**Figures 6–13.**
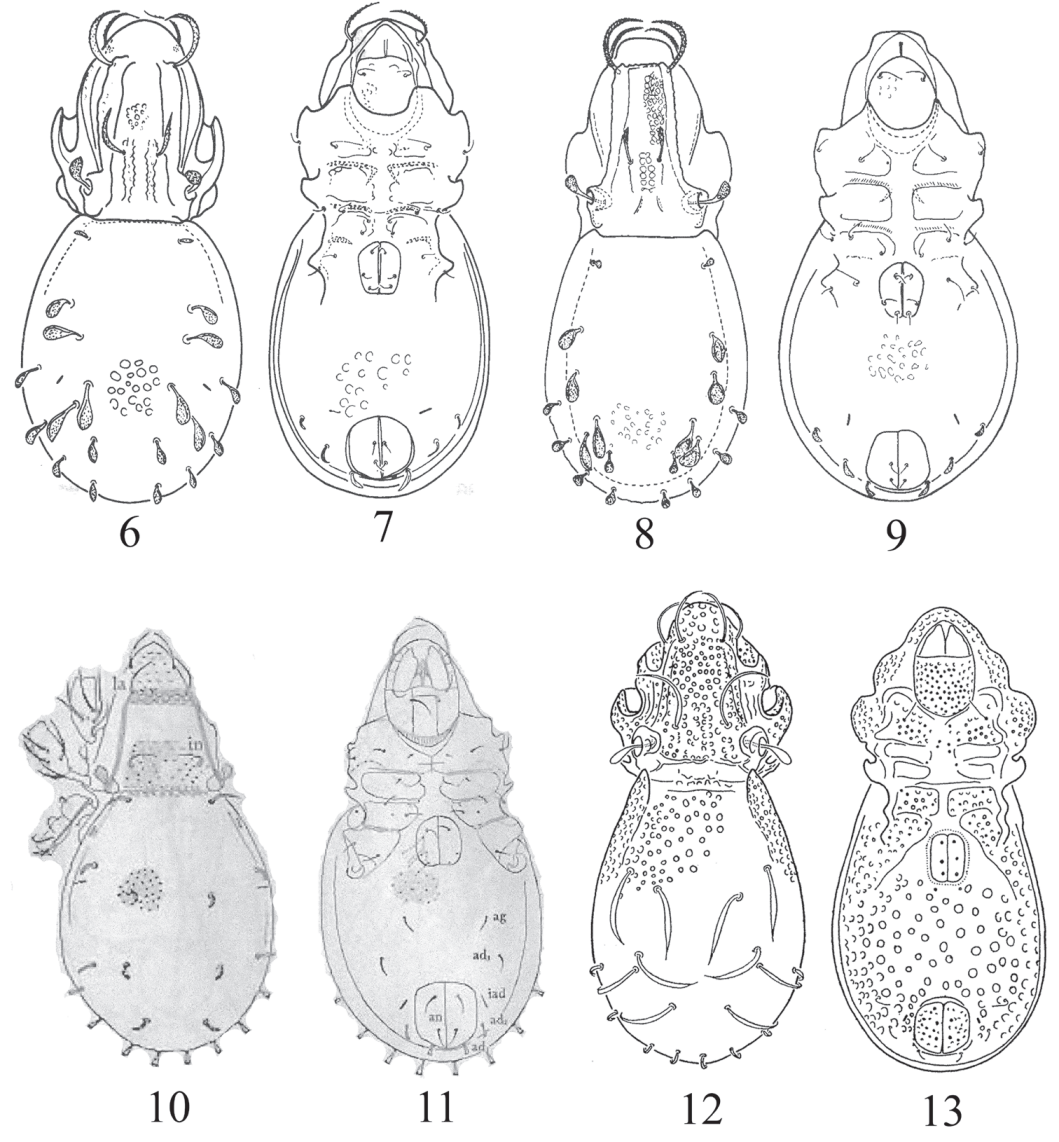
Species of the genus *Papillocepheus*, adult (**6, 8, 10, 12** dorsal view; **7, 9, 11, 13** ventral view): **6, 7** *Papillocepheus areolatus* Mahunka, 1987 **8, 9** *Papillocepheus decoratus* Mahunka, 1994 **10, 11** *Papillocepheus decorus* (Hammer, 1966) **12, 13** *Papillocepheus deficiens* J. & P. Balogh, 1983. Figures from: Mahunka 1987, 1994; Hammer 1966; Balogh and Balogh 1983, accordingly. Scale bars absent in original descriptions.

**Figures 14–23.**
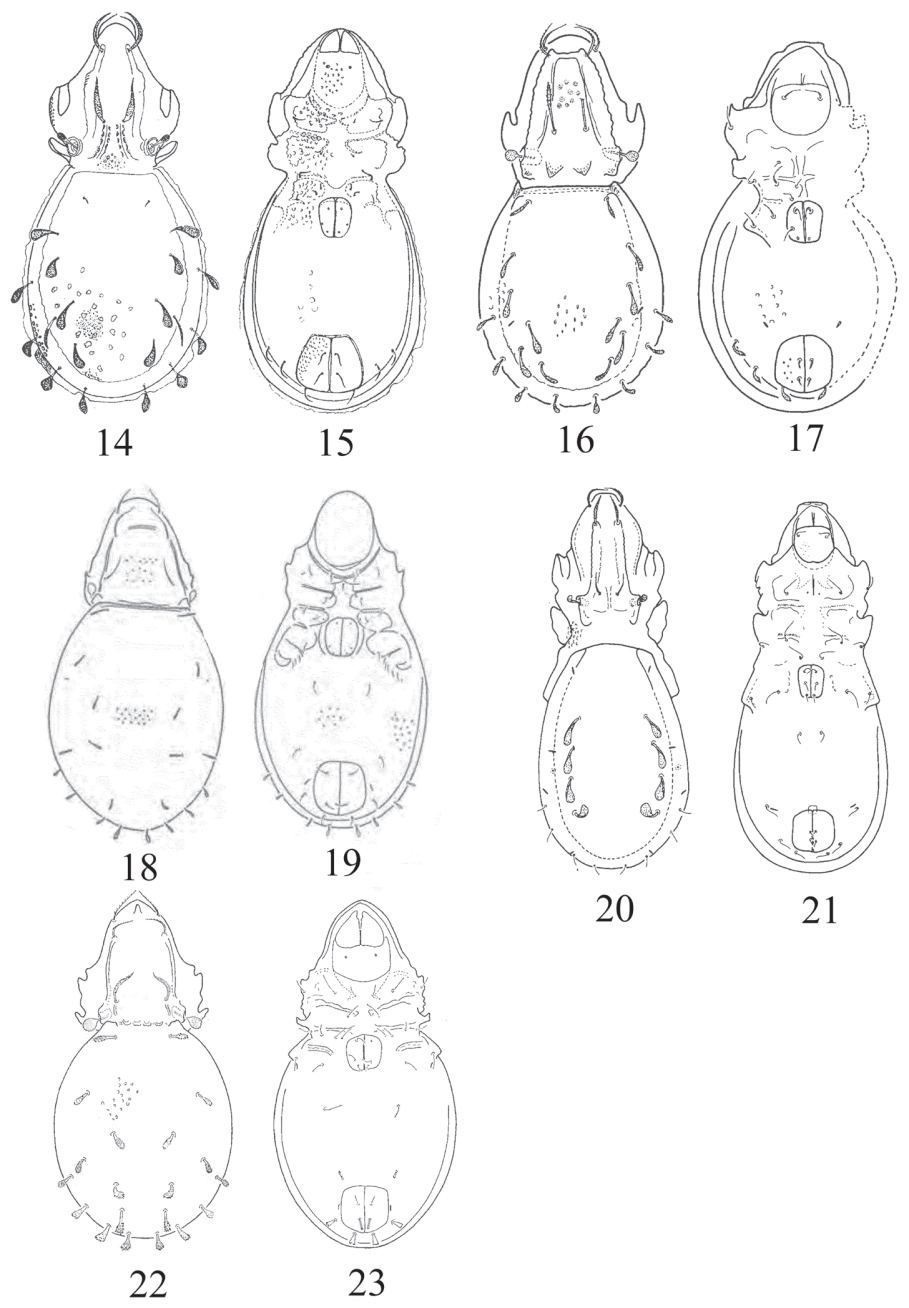
Species of the genus *Papillocepheus*, adult (**14, 16, 18, 20, 22** dorsal view; **15, 17, 19, 21, 23** ventral view): **14, 15** *Papillocepheus heterotrichus* Balogh & Mahunka, 1966 **16, 17** *Papillocepheus longisetosus* Mahunka, 2009 **18, 19** *Papillocepheus neotropicus* (P. Balogh, 1988) **20, 21** *Papillocepheus reductus* Mahunka, 1994 **22, 23** *Papillocepheus tuberculatus* (Mahunka, 1978). Figures from: Balogh and Mahunka 1966; Mahunka 2009; Balogh and Balogh 1988; Mahunka 1994, 1978, accordingly. Scale bars absent in original descriptions.

## Supplementary Material

XML Treatment for Papillocepheus

XML Treatment for Papillocepheus primus
